# Supplementary material for: Modelling protein complexes with crosslinking mass spectrometry and deep learning
Source: Nat Commun. 2024 Sep 9;15:7866. doi: 10.1038/s41467-024-51771-2 (PMC11383924; doi:10.1038/s41467-024-51771-2)
Supplement: Supplementary file 3 — Description of Additional Supplementary Files [file 41467_2024_51771_MOESM3_ESM.pdf]

File Name: Supplementary Movie 1

Description: Supplementary movie depicting the conformation change of Fur-Fpa
