# Supplementary material for: Modelling protein complexes with crosslinking mass spectrometry and deep learning
Source: Nat Commun. 2024 Sep 9;15:7866. doi: 10.1038/s41467-024-51771-2 (PMC11383924; doi:10.1038/s41467-024-51771-2)
Supplement: Supplementary file 6 — Source data [file 41467_2024_51771_MOESM6_ESM.zip › source data/source_data_supplement_figure13.pptx]

## Slide 1
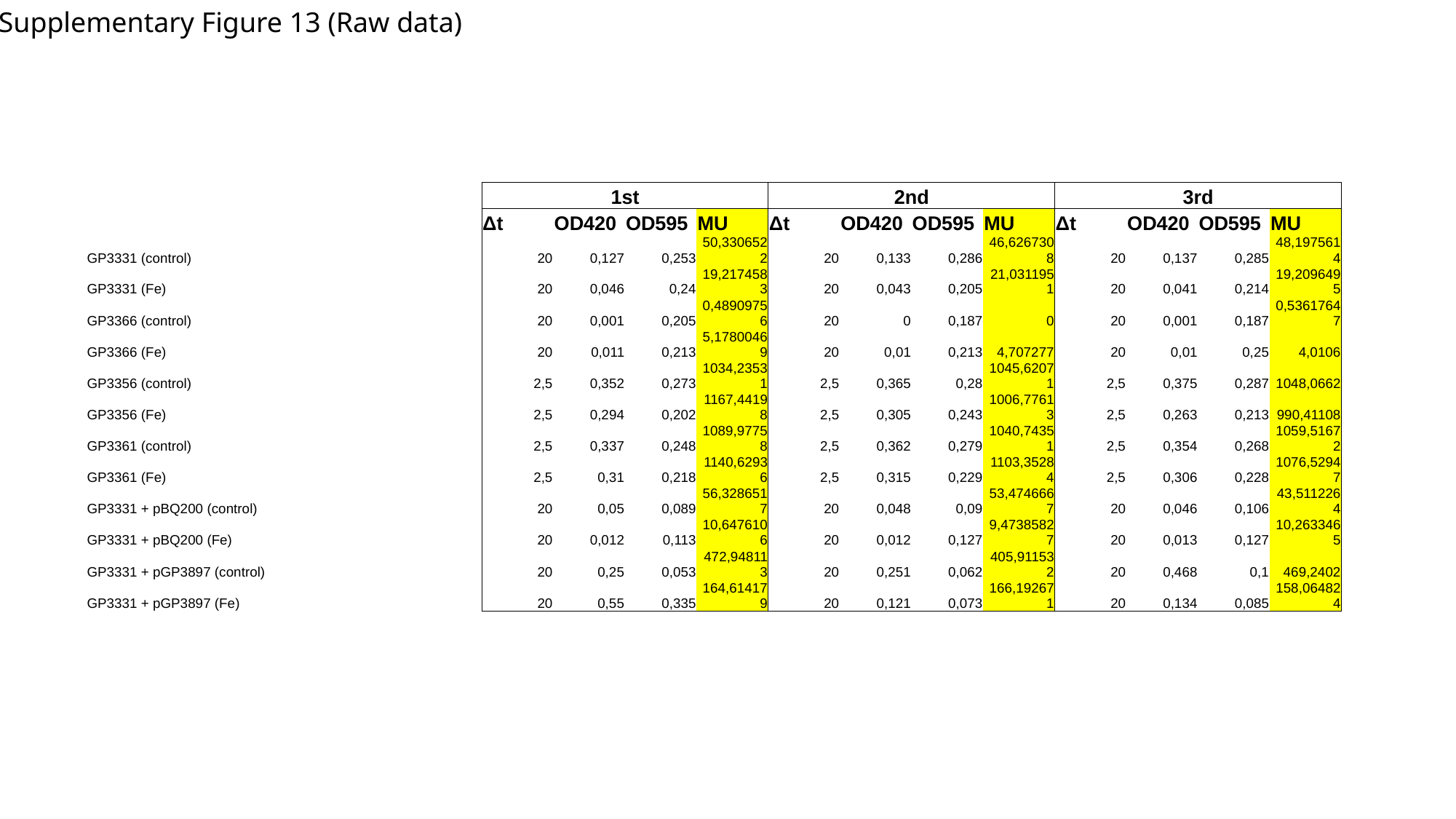

Supplementary Figure 13 (Raw data)
| | 1st | | | | 2nd | | | | 3rd | | | |
| --- | --- | --- | --- | --- | --- | --- | --- | --- | --- | --- | --- | --- |
| | Δt | OD420 | OD595 | MU | Δt | OD420 | OD595 | MU | Δt | OD420 | OD595 | MU |
| GP3331 (control) | 20 | 0,127 | 0,253 | 50,3306522 | 20 | 0,133 | 0,286 | 46,6267308 | 20 | 0,137 | 0,285 | 48,1975614 |
| GP3331 (Fe) | 20 | 0,046 | 0,24 | 19,2174583 | 20 | 0,043 | 0,205 | 21,0311951 | 20 | 0,041 | 0,214 | 19,2096495 |
| GP3366 (control) | 20 | 0,001 | 0,205 | 0,48909756 | 20 | 0 | 0,187 | 0 | 20 | 0,001 | 0,187 | 0,53617647 |
| GP3366 (Fe) | 20 | 0,011 | 0,213 | 5,17800469 | 20 | 0,01 | 0,213 | 4,707277 | 20 | 0,01 | 0,25 | 4,0106 |
| GP3356 (control) | 2,5 | 0,352 | 0,273 | 1034,23531 | 2,5 | 0,365 | 0,28 | 1045,62071 | 2,5 | 0,375 | 0,287 | 1048,0662 |
| GP3356 (Fe) | 2,5 | 0,294 | 0,202 | 1167,44198 | 2,5 | 0,305 | 0,243 | 1006,77613 | 2,5 | 0,263 | 0,213 | 990,41108 |
| GP3361 (control) | 2,5 | 0,337 | 0,248 | 1089,97758 | 2,5 | 0,362 | 0,279 | 1040,74351 | 2,5 | 0,354 | 0,268 | 1059,51672 |
| GP3361 (Fe) | 2,5 | 0,31 | 0,218 | 1140,62936 | 2,5 | 0,315 | 0,229 | 1103,35284 | 2,5 | 0,306 | 0,228 | 1076,52947 |
| GP3331 + pBQ200 (control) | 20 | 0,05 | 0,089 | 56,3286517 | 20 | 0,048 | 0,09 | 53,4746667 | 20 | 0,046 | 0,106 | 43,5112264 |
| GP3331 + pBQ200 (Fe) | 20 | 0,012 | 0,113 | 10,6476106 | 20 | 0,012 | 0,127 | 9,47385827 | 20 | 0,013 | 0,127 | 10,2633465 |
| GP3331 + pGP3897 (control) | 20 | 0,25 | 0,053 | 472,948113 | 20 | 0,251 | 0,062 | 405,911532 | 20 | 0,468 | 0,1 | 469,2402 |
| GP3331 + pGP3897 (Fe) | 20 | 0,55 | 0,335 | 164,614179 | 20 | 0,121 | 0,073 | 166,192671 | 20 | 0,134 | 0,085 | 158,064824 |
