# Supplementary material for: Modelling protein complexes with crosslinking mass spectrometry and deep learning
Source: Nat Commun. 2024 Sep 9;15:7866. doi: 10.1038/s41467-024-51771-2 (PMC11383924; doi:10.1038/s41467-024-51771-2)
Supplement: Supplementary file 6 — Source data [file 41467_2024_51771_MOESM6_ESM.zip › source data/source_data_supplement_figure15.pptx]

## Slide 1
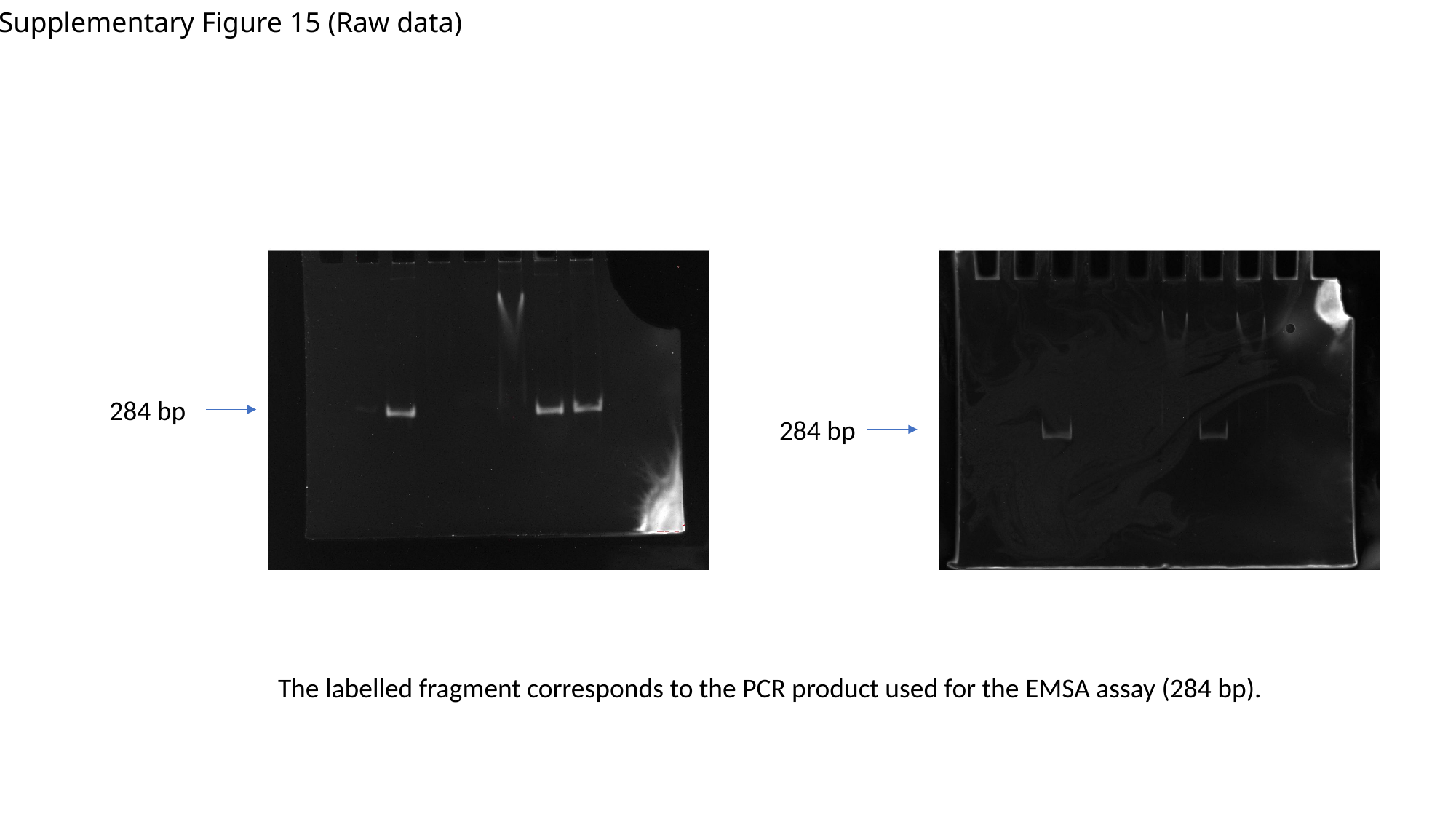

Supplementary Figure 15 (Raw data)
284 bp
284 bp
The labelled fragment corresponds to the PCR product used for the EMSA assay (284 bp).
