# Supplementary figures and images for: Modelling protein complexes with crosslinking mass spectrometry and deep learning
Source: Nat Commun. 2024 Sep 9;15:7866. doi: 10.1038/s41467-024-51771-2 (PMC11383924; doi:10.1038/s41467-024-51771-2)

## Slide 1
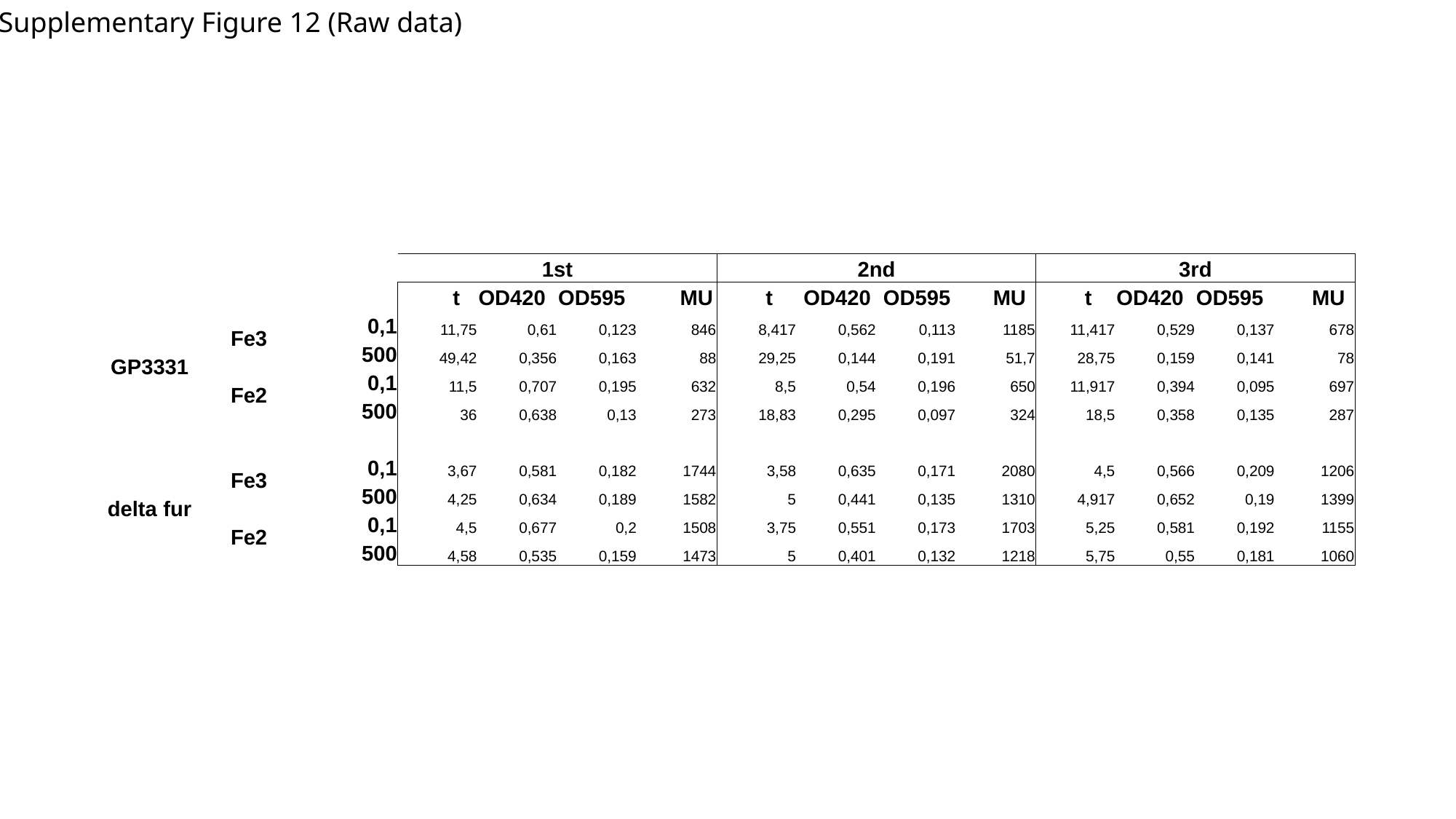

Supplementary Figure 12 (Raw data)
| | | | 1st | | | | 2nd | | | | 3rd | | | |
| --- | --- | --- | --- | --- | --- | --- | --- | --- | --- | --- | --- | --- | --- | --- |
| | | | t | OD420 | OD595 | MU | t | OD420 | OD595 | MU | t | OD420 | OD595 | MU |
| GP3331 | Fe3 | 0,1 | 11,75 | 0,61 | 0,123 | 846 | 8,417 | 0,562 | 0,113 | 1185 | 11,417 | 0,529 | 0,137 | 678 |
| | | 500 | 49,42 | 0,356 | 0,163 | 88 | 29,25 | 0,144 | 0,191 | 51,7 | 28,75 | 0,159 | 0,141 | 78 |
| | Fe2 | 0,1 | 11,5 | 0,707 | 0,195 | 632 | 8,5 | 0,54 | 0,196 | 650 | 11,917 | 0,394 | 0,095 | 697 |
| | | 500 | 36 | 0,638 | 0,13 | 273 | 18,83 | 0,295 | 0,097 | 324 | 18,5 | 0,358 | 0,135 | 287 |
| | | | | | | | | | | | | | | |
| delta fur | Fe3 | 0,1 | 3,67 | 0,581 | 0,182 | 1744 | 3,58 | 0,635 | 0,171 | 2080 | 4,5 | 0,566 | 0,209 | 1206 |
| | | 500 | 4,25 | 0,634 | 0,189 | 1582 | 5 | 0,441 | 0,135 | 1310 | 4,917 | 0,652 | 0,19 | 1399 |
| | Fe2 | 0,1 | 4,5 | 0,677 | 0,2 | 1508 | 3,75 | 0,551 | 0,173 | 1703 | 5,25 | 0,581 | 0,192 | 1155 |
| | | 500 | 4,58 | 0,535 | 0,159 | 1473 | 5 | 0,401 | 0,132 | 1218 | 5,75 | 0,55 | 0,181 | 1060 |

Supplement: Supplementary file 6 — Source data [file 41467_2024_51771_MOESM6_ESM.zip › source data/source_data_supplement_figure12.pptx]

## Slide 1
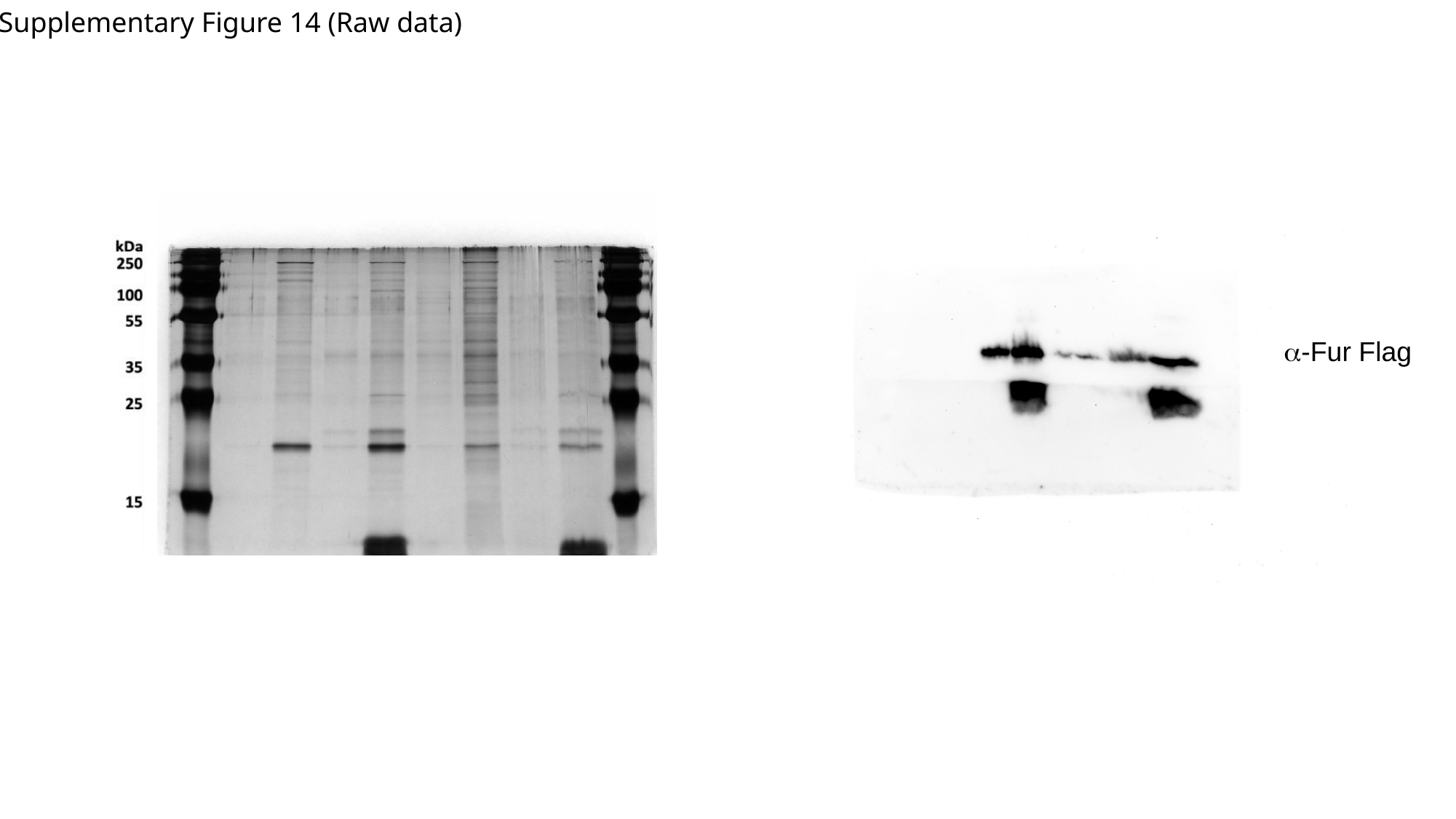

Supplementary Figure 14 (Raw data)
a-Fur Flag

Supplement: Supplementary file 6 — Source data [file 41467_2024_51771_MOESM6_ESM.zip › source data/source_data_supplement_figure14.pptx]
